# Reducing the ‘Silence Between Sessions’: A Qualitative Study on Youth and Professionals' Perspectives on Digital Tools for Suicide Prevention

**DOI:** 10.1111/hex.70572

**Published:** 2026-01-23

**Authors:** Elise Carrotte, India Bellairs‐Walsh, Sarah Hetrick, Jo Robinson, Eleanor Bailey

**Affiliations:** ^1^ Orygen, The National Centre of Excellence in Youth Mental Health Parkville Victoria Australia; ^2^ Centre for Youth Mental Health The University of Melbourne Parkville Victoria Australia; ^3^ Faculty of Medical and Health Sciences University of Auckland Auckland New Zealand

**Keywords:** digital health, digital intervention, qualitative research, self‐harm, suicide prevention, young people

## Abstract

**Introduction:**

Despite academic and clinical interest in digital suicide prevention tools (‘digital tools’), challenges persist related to their integration into existing care pathways. The objective of this study was to understand how young people and professionals use and perceive digital tools for the management of suicidal thoughts and/or self‐harming behaviours.

**Methods:**

This qualitative study involved interviews with young people aged 18–25 with lived experience of suicidal thoughts and/or self‐harming behaviours (*n* = 8), and with clinical or research expertise in youth suicide and digital interventions (*n* = 9). Interview transcripts were analysed using reflexive thematic analysis.

**Results:**

The analysis generated three main themes: (1) bridging the gaps in support, (2) user experience while navigating suicidal thoughts or crisis, and (3) digital tool implementation: expectation versus reality. Further, sub‐themes were: (i) unique suicide prevention opportunities inherent to digital tools, (ii) complexity of digital risk management, (iii) privacy considerations around a stigmatised topic, and (iv) (perceived) low uptake by young people.

**Conclusion:**

Digital tools that are user‐friendly, adaptable, and personalised may enable tailored and timely support for the prevention and management of suicide and self‐harm. Nonetheless, poor user experience, organisational buy‐in, and difficulties managing suicide risk data outside clinical environments pose challenges to effective implementation, scale‐up, and sustained engagement. Participatory methods, such as co‐design, may help address these issues.

**Patient or Public Contribution:**

Young people with lived experience of suicidal thoughts and/or self‐harming behaviours, and who had used digital tools for suicide prevention purposes, were participants in this study. Study processes were reviewed by Orygen's Youth Research Council prior to study commencement.

## 1 Introduction

Despite suicide being a leading cause of death for young people in many countries [1], it is estimated that worldwide, less than half of young people who experience suicidal thoughts and/or self‐harming behaviours (STBs) receive treatment [2]. Major barriers to care for young people include access to timely, affordable and appropriate support [3, 4], the stigma associated with disclosing and seeking support for STBs [4, 5], and a desire to self‐manage distress [6].

One potential solution involves digital suicide prevention tools (‘digital tools’). Digital tools are client or patient‐facing tools that comprise any application or tool that utilises a digital interface or features, and include mobile apps, websites, online forums, therapeutic games, and other digital platforms [7]. Digital tools that are targeted toward users experiencing STBs incorporate various strategies, including safety planning (including integration with support from family and friends); facilitating access to digital peer support; psychoeducation and therapeutic techniques, including homework tasks; crisis management (including facilitating contact with crisis support services); and outreach or follow‐up strategies for people who have self‐harmed or made a suicide attempt [8, 9]. Some are designed for integration with clinical care via ‘hybrid’ use, and may involve digitisation of existing therapeutic strategies, allowing an opportunity to transform evidence‐based therapies (such as cognitive behavioural therapy) into a digital setting [10, 11]. Other tools are fully autonomous and may be utilised in a self‐guided manner, without recommendation or guidance from a health professional [10, 12].

Digital tools demonstrate significant potential for preventing and managing STBs, particularly for young people. Adolescents and young adults are more likely to embrace digital tools to manage STBs compared to older people, and report high rates of access to the Internet and smartphone technologies [13]. Promisingly, young people typically rate digital tools for STBs as acceptable and minimally burdensome [14, 15]. From the service provider perspective, digital tools also appear beneficial, as they may inform the quality and comprehensiveness of assessments, improve treatment planning and post‐discharge check‐ins, and facilitate personalised care [16, 17].

Consequently, in recent years, more attention has been paid to the role of digital tools specifically designed for people experiencing STBs. Smartphone apps [8, 11] and web‐based tools such as online psychoeducation and therapeutic modules [18, 19] have received the most attention from researchers and clinicians. However, digital tools targeting STBs are less studied compared to those targeting conditions such as depression, anxiety, stress, PTSD, and eating disorders [20, 21], though such tools may incorporate suicide risk screening and crisis support pathways.

To date, systematic reviews and meta‐analyses consistently find that digital tools show small but significant reductions in suicidal ideation in young people compared to control groups [14, 22, 23, 24]. There is less evidence demonstrating the impacts of digital tools on reducing rates of suicide attempts, compared to suicidal ideation [24]. Findings are most promising for digital tools incorporating cognitive behavioural therapy, dialectical behaviour therapy, problem‐solving strategies, and safety planning features, for hybrid use, and for those that directly target suicidal ideation [14, 22, 24].

However, implementation challenges are prevalent regarding digital tools. Studies have described attrition when using digital tools in real‐life settings; for example, it is estimated that only 3.3% of users are still using popular digital mental health apps within 30 days of download [25]. Another study, specific to the context of STBs, found that only one‐third of young people report accessing their digital safety plans during high‐risk periods [26]. Of concern, many mental health apps on the market also lack evidence, with one study estimating that only 3% of apps have research justifying claims of effectiveness, though one‐third claim to have expert mental health development input [27].

There is a need to deepen our understanding of how digital tools are being used for young people experiencing STBs, including types of tools, their perceived benefits, and facilitators and barriers to real‐world uptake. Notably, young people and professionals may have different preferences and perspectives regarding what constitutes useful and acceptable digital tools, and what value they may derive from either standalone or hybrid use [28]. Hence, there is also a need to integrate the perspectives of clinicians and other professionals involved in the design, implementation, and evaluation of such digital tools. Such findings can inform future design and implementation efforts. Hence, the objective of this study was to understand how young people with lived experience, researchers and healthcare professionals, use and perceive digital tools for the management of suicidal thoughts and/or self‐harming behaviours.

## 2 Materials and Methods

### 2.1 Study Design

This was a qualitative study comprising individual interviews, approved by the University of Melbourne Human Research Ethics Committee (approval number: 2024‐14236‐58858‐8). The interviews were originally conducted as part of a larger Delphi consensus study [7] that led to the development of guidelines for integrating digital tools into clinical care for young people experiencing STBs. Early findings from the qualitative interviews were originally used to generate and inform items included in the Delphi consensus study [7]; however, interview data had not previously been analysed thematically.

### 2.2 Participants and Recruitment

Young people were recruited through social media advertising, and were eligible if they were (A) aged 15–25 years old, (B) had lived experience of using digital tools to manage STBs, (C) lived in Australia and (D) reported no or infrequent suicidal thoughts in the past 2 weeks. Australian residency (criterion C) was required to support adequate and timely referral and safety escalation procedures in the event that an interview participant disclosed current psychological distress or suicidal ideation requiring immediate support. Criterion D was assessed through question 9 on the Patient Health Questionnaire (PHQ‐9) [29], which was adapted to allow participants with recent infrequent or fleeting suicidal thoughts to participate. Young people received AUD $30 per interview.

Professionals were individuals with clinical or research expertise in digital interventions and youth suicide prevention. They were identified through purposive sampling by the research team, based on professional networks and relevant publication history, and invited to participate via email. Inclusion criteria for professionals were: (a) current employment in a clinical service setting with experience working with young people experiencing STBs, either in a client‐facing or managerial role; or (b) publication of research on digital interventions for suicide prevention; and (c) residence in a predominantly English‐speaking country. Professionals were not renumerated for interviews.

### 2.3 Procedure

All participants provided informed consent through viewing and signing a digital consent form. Participants attended semi‐structured interviews conducted via Zoom during the Australian COVID‐19 lockdowns in 2020. Interviews were audio‐recorded and transcribed by a professional transcription service. Interviews were conducted by authors EB and IBW, experienced researchers in suicide prevention.

The discussion guide is included as an Appendix; separate questions were drafted for healthcare professionals and researchers, though if participants were employed in both roles, they were asked a combination of questions relevant to their expertise. Interviews with young people included brief demographic questions and questions on participants' experience of using digital tools for the management of STBs. Interviews with professionals included questions about their experiences using digital tools as part of clinical practice and their views regarding barriers and facilitators to the implementation of digital tools.

### 2.4 Data Analysis

A reflexive thematic analysis was undertaken on transcribed data using the methodology outlined by Braun and Clarke [30]. The analysis was led by author EC, who is a female clinician‐researcher in suicide prevention and has clinical experience integrating digital tools into practice, but not specifically for suicide prevention. EC has also been employed in a clinical governance role for a digital mental health service, and has experience writing safety management protocols for digital collection of suicide‐related data through ecological momentary assessment methodologies [31].

To begin analysis, EC carefully read and re‐read all transcripts and generated codes using a critical realist approach [32]: a philosophical approach to qualitative research which acknowledges the presence of reality or ultimate truth, but acknowledges that subjective researcher and participant beliefs influence their interpretation of this reality. Coding aimed to examine meaning at the underlying or implicit level, as well as recognising themes and ideas that are more explicit or objective. This process was primarily inductive, where codes were generated based on patterns identified by EC through the process of reading and re‐reading transcripts. Some deductive coding also occurred based on expected themes identified in prior literature, such as early code labels relating to ‘user experience’ [33, 34, 35] and suicide‐related ‘risk management’ [36, 37]. Early codes were organised into candidate themes and sub‐themes via NVivo v14 software, which were refined through discussion with IBW to develop a shared interpretation of the data. Themes and sub‐themes were further refined through discussion with the broader research team prior to finalisation.

## 3 Results

Eight young people and nine professionals participated in the study. Young people were aged 18–25 years, with a mean age of 22.1 years (SD = 2.7); all were born and living in Australia. Other demographic details for the youth sample are presented in Table 1. Among the professional sample, there were four clinicians, four researchers, and one clinician‐researcher. Countries of residence were Australia (*n* = 3), New Zealand (*n* = 3), England (*n* = 2) and the Netherlands (*n* = 1). Mean interview length was 48.5 min for young people and 34.4 min for professionals.

**Table 1:** Demographic characteristics of the youth sample (*n* = 8).

Variable	*n*	%
Gender		
Female	4	50%
Male	2	25%
Trans male	1	13%
Gender queer	1	13%
Cultural background		
White Australian	5	63%
Malaysian/Vietnamese	1	13%
Malaysian/Australian	1	13%
Indian	1	13%
Sexual orientation		
Heterosexual/straight	0	0%
Gay/homosexual	0	0%
Bisexual	6	75%
Pansexual	1	13%
Prefer not to say	1	13%
Religion		
Christian	3	38%
Sikh	1	13%
Spiritual	1	13%
Agnostic or not religious	3	38%
Highest level of education		
Year 11	1	13%
Year 12	4	50%
Diploma	1	13%
Undergraduate degree	1	13%
Postgraduate degree	1	13%
Location		
Metropolitan	4	50%
Urban	3	38%
Remote	1	13%
Employment		
Employed	3	38%
Unemployed and/or government subsidy	4	50%
Not reported	1	13%

**Figure 1:**
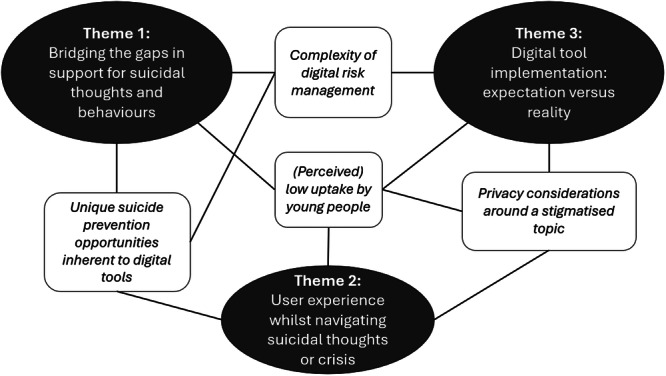
Thematic map.

The reflexive thematic analysis generated three overarching themes and four sub‐themes. Themes and sub‐themes were closely linked together; they are visualised in the thematic map presented in Figure 1.

### 3.1 Theme 1: Bridging the Gaps in Support for Suicidal Thoughts and Behaviours

Young people reported accessing a wide range of digital tools for management of STBs, including psychoeducational materials (e.g., fact sheets, YouTube videos), mindfulness and meditation apps, mood‐tracking tools, informal peer forums (e.g., Reddit), and professional services such as online counselling. Although many tools were self‐directed, some were recommended by mental health professionals and woven into face‐to‐face care. Most digital tools used by young people focused on general wellbeing or mental health rather than STBs specifically, and usage was often exploratory and temporary, driven by immediate needs and perceived usefulness. Reflecting on this, one participant described a process of trial and error:

‘I have experimented with a couple of different apps, like mobile apps … like the general strategies, like input a wellness plan and just have some like tips to go to. If you were feeling in distress, you will be able to follow those strategies … Sometimes meditation and breathing exercises have been really good … but there is online web‐based things like online support forums that are really good. That I've used for self‐harm and suicide’

(P3, young person).

Accessibility was identified as a key benefit of digital tools. Participants reported encountering difficulties finding an appropriate in‐person service, significant out‐of‐pocket costs, and service disruptions during events such as COVID‐19 lockdowns. In this context, having support ‘in your pocket’ provided an immediate alternative and was a ‘godsend’ when traditional care was unavailable (P3, young person), such as in the context of long waitlists:

‘I've spoken to young people who have been on waiting lists for two years plus. By the time they get seen, they're being seen in adult services, so it's not as if they even get there. I mean, in terms of risk, at that point … there's no management of risk’

(P9, professional).

Digital tools also bridged a gap for those not requiring crisis intervention but needing more support than periodic professional support, reducing the ‘silence between sessions’ (P3, young person). Digital tools could also provide an alternative entry point to mental healthcare; one young person observed, ‘doing a digital chat is way less confronting … there's not as much pressure and you're able to say some things that might be harder to say out loud’ (P1, young person).

Despite these advantages, participants agreed that digital tools are not a panacea. While some platforms were designed with safety features in mind (e.g., trained moderators, emergency support pathways), others lacked such features. Informal peer forums posed additional risks: exposure to unvetted advice, harmful practices, or ‘triggering’ content that could exacerbate distress (P2, young person). Some participants observed that many wellbeing‐focused digital tools are frequently utilised by individuals experiencing acute or chronic STBs – populations for whom these tools were neither originally designed nor adequately supported:

‘We're seeing more and more that [service users who] have experienced complex mental health conditions, longstanding suicidality, or high psychosocial complexity … are opting for digital services’

(P16, professional).

Many participants, both young people and professionals, emphasised that digital tools ideally should complement, rather than replace, clinical care. Some believed that these tools ‘will never replace real in‐person relationships and being able to speak to someone’, as the therapeutic alliance is ‘intangible’ (P3, young person). Digital tools integrated into therapy, such as app‐based homework assignments or clinician‐reviewed mood logs, were generally more appealing to practitioners as opposed to standalone digital tools:

‘They enable resources to be used more effectively, or they blend into what the therapist is already doing and act as an extension of the intervention’

(P9, professional).

Others argued that well‐designed digital interventions deserve recognition as standalone, beneficial treatments. One professional asserted that ‘digital is often seen as an adjunct, where I think it has a status in its own right’ (P16, professional), urging a shift beyond viewing these platforms as secondary supports for young people experiencing STBs.

#### 3.1.1 Unique Suicide Prevention Opportunities Inherent to Digital Tools

Participants identified several features unique to digital tools that extend beyond traditional face‐to‐face services. ‘Ongoing accurate record‐keeping’ (P12, professional) of mood and STBs was highlighted as a key advantage. One young person noted that a digital tool is beneficial as it ‘keeps track over time … I might remember bits and pieces, but I'm not going to remember the past four weeks’ (P6, young person) when clinical support sessions are infrequent.

Participants noted that safety planning modules, psychoeducational content on underlying issues (e.g., mood difficulties, anxiety, substance use), and coping skill tutorials could be integrated into digital tools, prompting users to identify patterns and engage with relevant strategies in a timely fashion. Automated crisis support features – such as in‐app prompts to deploy distress tolerance techniques, or ‘panic button’ (P11, professional) shortcuts to emergency services – were seen as vital for addressing spikes in distress, particularly during late‐night hours when professional help is often inaccessible. Such features could also foster a young person's understanding of their STBs, including their triggers, as one professional described:

‘At crisis point … the main role [of digital tools] would be to guide them to resources to help them improve their understanding of their behaviours and why they're doing it … and then maybe bring in other monitoring tools in between sessions to see how they've been over that time’

(P14, professional).

### 3.2 Theme 2: User Experience While Navigating Suicidal Thoughts or Crisis

Participants emphasised that user experience is critical for the success of digital tools, especially during the complex experience of STBs, ideally ‘user friendly both for the client but also for the clinician’ (P13, professional). Tools must appeal to young people and match their therapeutic goals; for example, some participants wanted tools with broad mental health‐related content, but others preferred features specific to the management of STBs. Participants agreed they must also fit clinicians' workflows and digital literacy. Opinions varied on the utility of building new platforms versus optimising existing ones, with some critical about creating new platforms: ‘We should understand what young people are using … It's no use developing a whiz‐bang platform if [their] main kind of social feeds are going to be Facebook, Instagram, and TikTok’ (P16, professional).

Intuitive features, engaging visuals, and youth‐friendly language were highlighted as essential: ‘If something has a good infographic, or it's just nicely laid out … I actually read it and pay attention, and usually I remember [content] better’ (P6, young person). Features like mood monitoring required simple data export and cross‐device access. For STB‐specific tools, emergency escalation features needed minimal text to avoid ‘overload[ing] people with information … you're kind of [in] that fight‐or‐flight response’ (P15, professional). Interactive safety planning prompts were considered useful if straightforward:

‘Don't get people following fancy plans and blah, blah, blah. Keep it only focused on reducing acute risk’

(P16, professional).

In order to embed good user experience into digital tools, participants strongly supported co‐design with those who have lived experience of STBs, alongside clinicians, families, and developers. Genuine involvement was deemed ‘essential’ (P14, professional), but many felt previous efforts were insufficient. ‘Many existing digital tools are not co‐designed very well’, said P3 (young person), who added:

‘I don't think that many organisations are willing to dedicate the amount of resources to properly co‐design or to properly train their staff and also train the young people … It feels very, very tokenistic, in the sense’

(P3, young person).

#### 3.2.1 Privacy Considerations Around a Stigmatised Topic

Security and confidentiality were described as ‘paramount’ (P14, professional) to digital tools. These concerns were especially salient for individuals in unsupportive or unsafe environments, those grappling with guilt or shame relating to their experience of STBs, or those with intersecting marginalised identities, such as LGBTIQA+ young people. Young people feared data breaches and expressed concerns about who might access their information; as P3 (young person) noted: ‘If you give [young people] a platform to actually write things down, it might not be safe … devices are more easily hacked nowadays’.

Young participants desired clear, simple explanations of digital tools' privacy policies and data sharing procedures, and influence over how and when their data may be shared. Many did not read the terms and conditions in detail, leading to unexpected scenarios. P1 (young person) recounted a ‘jarring’ experience visiting a mental health professional working in the same organisation that hosted a webchat service they had previously accessed. The clinician raised content of the old webchat during their in‐person session:

‘[The mental health professional said], “Oh yeah so this is what you talked about in [digital chat]…” I'm like, “No, not really,” because … the reason I was using [the digital chat] in the first place was this really shameful thing…’

(P1, young person).

Another young person, P2, described being located by police after disclosing suicidal thoughts in a crisis service webchat they had perceived to be anonymous. Though initially shocked, they later felt ‘really grateful’ as this interaction allowed them to stay safe.

### 3.3 Theme 3: Digital Tool Implementation: Expectation Versus Reality

Despite sector interest and eagerness, professionals identified numerous obstacles to integrating digital tools into routine practice. While some digital tools – such as mindfulness and meditation apps – are normalised as adjuncts to therapy, digital suicide prevention tools often struggle to gain traction. As one professional observed:

‘One of the biggest problems with digital interventions is that even when they have an amazing evidence base, the chances of them being implemented and sustained are very, very slim’

(P9, professional).

Both young people and professionals reported implementation challenges at individual and organisational levels. Clinicians noted that they often lack time or resourcing to learn new platforms, making training – often seen as a ‘luxury’ (P16, professional) – hard to prioritise. One young person noted that clinician buy‐in is crucial: ‘You're not really going to get a young person on board with something that their therapist isn't genuinely on board with themselves’ (P3, young person).

Sustainability and ongoing costs were cited repeatedly, especially for digital tools requiring moderation or clinician governance: ‘you start getting into shift, roster work, loadings … it costs more than your face‐to‐face services’ (P16, professional). As one professional explained,

‘It's all good and well to say we're going to offer these online technologies. But if you don't have the infrastructure or resourcing in your organisation to do that, then what?’

(P12, professional).

#### 3.3.1 Complexity of Digital Risk Management

Professionals acknowledged that identifying STBs through digital tools adds complexity to clinical care. Paradoxically, some professionals described a culture of clinicians and researchers preferring not to gain a fuller picture of young people's STBs through digital tools, to avoid having to implement or adapt risk management procedures in response:

‘We had so many people just saying, “oh, no, can we just remove those risk questions [from the digital tool]…?” But this is bizarre. So, avoiding asking those questions somehow makes them okay and you don't do anything’

(P9, professional).

Collecting real‐time data about STBs raises issues related to duty of care and 24/7 availability: ‘Once you identify that someone is at risk, you have to do something’ (P9, professional). Concerns about legal and ethical implications as a result were raised:

‘If you refer a client to an app or a tool and they're suicidal and they die by suicide what's the legal implication … Are you going to be framed as someone who's neglected their client and handed them off to a computer?’

(P12, professional).

When STB monitoring data is collected, clear boundaries and expectations were described as essential. Clinicians stressed the need to communicate with young people how and when they review data, what constitutes an emergency, and the limits of their availability.

Participants also warned against overly sensitive algorithms that trigger safety protocols despite a young person being safe. As P9 (professional) explained, ‘Just because someone is feeling suicidal doesn't mean that's a high risk. Quite a lot of young people might feel like that consistently … but that's something they live with’. Participants advocated for user autonomy in flagging serious concerns:

‘At some point, we should trust them … they should have the autonomy to share without thinking, “Everyone's going to overreact and I'm going to end up in hospital again.”’

(P9, professional).

#### 3.3.2 (Perceived) Low Uptake by Young People

Despite the proliferation of digital tools, professionals observed that many young clients never use tools, or abandon tools soon after trialling them, leading to clinician disillusionment and ‘lost enthusiasm’ (P10, professional). However, young people highlighted practical barriers to ongoing use, particularly in the app context; barriers included user experience challenges, Internet and device issues, and feeling overwhelmed by choice and uncertain which tools to trust: ‘There are so many resources … you almost get lost in it’ (P7, young person). Parent and family scepticism also influenced uptake; as P12 (professional) noted, ‘There's suspicion by parents that perhaps doing things online isn't real therapy either’.

Some young people noted that maintaining motivation for ongoing use was challenging: ‘as someone who has struggled with depression, I get home and then have no motivation to [use the tool], even though I know it's helpful’ (P6, young person). Negative past experiences, including long waits for certain crisis‐specific digital services and unsatisfactory interactions, fostered mistrust.

Recognising diverse needs, participants stressed that no single digital tool suits everyone. They emphasised the need to match tools to individual recovery stages, co‐occurring conditions, and personal goals, and in some cases, requiring ‘a bit of trial and error’ (P12, professional). Shared decision‐making and giving young people autonomy to choose and trial different digital tools were recommended strategies to boost engagement:

‘Making sure that the client is able to pick different [tools], making sure that they have the autonomy to actually pick which one they like and have a trial run’

(P15, professional).

## 4 Discussion

This qualitative study aimed to understand how young people with lived experience and professionals use and perceive a variety of digital tools for the management of STBs. Through in‐depth qualitative interviews with young people and professionals, this study identified the potential of digital tools to bridge the gaps in young people's access to support for STBs by reducing barriers associated with in‐person supports and unique digital features. However, user experience considerations were paramount to the usefulness of digital tools, particularly during a suicidal crisis, and implementation challenges were highlighted.

Young people emphasised the value of digital tools in providing interim support in between sessions with professional services, and increasing access to support when they might be unable or unwilling to seek professional support due to service coverage issues, or the stigma associated with suicide, aligning with several prior studies [3, 4, 5]. Young people discussed trialling digital tools to manage their STBs, sometimes in the context of mental health concerns, in both a hybrid manner (integrated with professional support) and as a standalone, self‐guided manner. This reflects trends in youth interest in self‐management of STBs [6] as well as the role of standalone tools, for which meta‐analyses report statistically significant improvements in STBs [11]. Furthermore, many young people reported using digital tools that were not STB‐specific; this indicates that they may not be accessing STB‐specific features such as safety planning. Regardless, transdiagnostic digital tools can be as beneficial as those that are disorder or topic‐specific [38].

Furthermore, the technological capacity of such tools was appealing to many young participants, especially those with the ability to track data pertaining to mood and STB‐related thoughts, urges, or behaviours. Considering the limited predictive validity of traditional suicide risk assessments [39], such data can support a nuanced, dynamic understanding of a young person's STBs, and potentially prompt young people to engage in coping strategies or even trigger timely, individualised interventions [16].

Yet professionals interviewed in the study emphasised that collecting such data results in a double bind: it requires clear, timely, and appropriate safety management procedures and clinical governance built within digital tools, to maintain their duty of care. This may reflect challenges identified in prior literature relating to a lack of clinician confidence around how to approach the nuances and ambiguities of risk assessments [37]. Clinicians do not consistently enquire about suicide [37] and have reported fear and discomfort regarding the accuracy of risk assessments, including fears of professional or legal ramifications associated with failing to adequately address suicide risk [36]. Regardless, the use of digital tools to monitor STBs raises important ethical questions about *how much* risk data should be collected, *when*, and *how* it should trigger safety escalation protocols, and how to balance this with young people's informed consent and autonomy.

Professionals emphasised that even when evidence is available for the effectiveness of digital tools, and they are interested, motivated, and able to incorporate these into their clinical care, implementation barriers persist. These barriers include poor fit with clinical workflows, resourcing costs, and lack of access to appropriate training and infrastructure, which are known barriers for implementation in suicide prevention more broadly [40]. Ongoing staff training, clear communication, operational support, support from senior staff, and reminders may be necessary for the successful integration of digital tools [41].

Clinicians also expressed frustration that young people do not consistently use tools that have been introduced into treatment. Yet young people describing a range of complex barriers to meaningful, long‐term usage aligned with previous literature that highlights confidentiality and privacy concerns, user experience difficulties, and a lack of personalisation, supporting prior research [33, 34, 35]. Technological barriers (e.g., lack of Internet access and device‐related issues) were also identified, which challenge the assumption that digital mental health tools are universally accessible to young people, and highlight the importance of digital inclusion as a social determinant of mental health [42].

Several participants emphasised that meaningful co‐design with young people, clinicians, and other professionals is an important strategy to make digital tools youth‐friendly and potentially improve acceptability and real‐world uptake [40]. Participatory research is gaining traction in both mental health and suicide prevention, including with young people [43, 44]. To date, however, most digital tools do not report any end‐user involvement in their design, and evaluation of co‐design processes is inconsistent and often low quality [44, 45]. Participatory methods should also be applied to implementation frameworks in order to increase sustainability and traction in real‐world settings [46].

The findings of this study have policy implications. They broadly support Australia's National Suicide Prevention Strategy 2025–2035 [47] recommends expanding the availability of both therapist‐supported and self‐guided digital tools to help overcome access barriers, especially for people in rural, regional, and remote Australia, and to identify and respond to suicidal distress (recommended actions 7.1f and 7.2c). This goal further aligns with Australia's Digital Health Blueprint 2023–2033 [48], which outlines the Australian Government's vision for safe, trustworthy, and effective digital health.

However, the findings also highlight the need for clear guidance for clinicians and organisations in terms of selecting digital tools with sufficient evidence and governance. Accreditation with the National Safety and Quality Digital Mental Health Standards [49] may assist clinicians in selecting tools that have been rigorously reviewed to meet standards regarding data privacy and security, safety management, and continuous improvement [47]. Decisions can be further supported by guidelines published by Bailey et al. [7] that aim to assist clinician decision‐making when incorporating digital tools into the care of young people experiencing STBs. These guidelines emphasise developing knowledge and awareness about the efficacy and effectiveness of digital tools for STBs; selecting tools based on quality, desired outcomes and their existing technology use; clear communication with the young person about the tool's purpose and boundary‐setting; and developing standardised processes for identifying and managing STBs through digital tools.

### 4.1 Strengths and Limitations

A strength of this study is that it integrates the perspectives of community‐based young people with lived experience of STBs with those of professionals; most prior studies on this topic focus on one perspective only, such as adolescents [33], or present findings for specific settings that do not reflect the breadth of digital tool usage, such as focussing solely on hospital settings [41].

A limitation is that the data are 5 years old at the time of writing. Regardless, many facilitators and barriers identified in this study are still highly relevant; notably, suicide rates in Australia have not decreased [50], and service accessibility is still a major concern. Also, all participants were familiar with digital tools from a variety of user, researcher, and clinician perspectives. Consequently, the analysis could not provide insights into barriers to use for those who have been unwilling to try digital tools. This study also covered a wide range of digital tools for management of STBs and was unable to explore specific benefits and challenges associated with all subtypes (e.g., apps vs. peer support forums).

## 5 Future Directions

Future research would benefit from exploring user and clinician views on technological innovations, including the influx of artificial intelligence (AI)‐based interventions [51, 52] and how these might apply to digital suicide prevention tools. Although guidance exists for young people in terms of navigating suicide‐related content on social media and online communities [53], similar guidance would be beneficial for choosing digital suicide prevention tools, including those with AI features. Researchers and clinicians developing new digital suicide prevention tools should also incorporate participatory methods, where possible [43, 44].

## 6 Conclusions

This study highlights the nuanced role digital tools can play in supporting young people experiencing STBs. Although participants recognised the potential benefits of digital tools, their real‐world uptake and effectiveness depend on meaningful design and thoughtful implementation strategies. Tools must be embedded within systems that support both user autonomy and clinicians' duty of care for safety concerns; they must also be designed with care regarding usability, privacy, and trust. Importantly, digital tools should be considered just one part of a broader, youth‐centred approach to suicide prevention and mental health support.

## Author Contributions

**Elise Carrotte:** formal analysis, writing – original draft, writing – review and editing. **India Bellairs‐Walsh:** formal analysis, investigation, writing – review and editing. **Sarah Hetrick:** conceptualisation, methodology, writing – review and editing. **Jo Robinson:** conceptualisation, funding acquisition, methodology, supervision, writing – review and editing. **Eleanor Bailey:** conceptualisation, funding acquisition, investigation, methodology, supervision, writing – review and editing.

## Ethics Statement

This study was approved by the University of Melbourne Human Research Ethics Committee (approval number: 2024‐14236‐58858‐8).

## Conflicts of Interest

The authors declare no conflicts of interest.

## Data Availability

Supporting data are not available, as the participants of this study did not give written consent for their data to be shared publicly, and the data could compromise the privacy of research participants.
